# Xanthoxylin Protects Against Alcoholic Liver Injury via the EGFR/AKT Pathway: A Combined In Silico and In Vitro Study

**DOI:** 10.3390/molecules31183230

**Published:** 2026-09-12

**Authors:** Xuanyou Li, Yiquan Lan, Chaoyi Xue, Keguang Yang, Lei He, Jun Sheng, Jing Wang, Peiyuan Sun

**Affiliations:** 1 Key Laboratory of Development and Utilization of Food and Medicinal Resources, Ministry of Education, Yunnan Agricultural University, Kunming 650201, China; lxuanyoug@163.com (X.L.); xuechaoyi@ynau.edu.cn (C.X.); 2 College of Food Science and Technology, Yunnan Agricultural University, Kunming 650201, China; 3 College of Science, Yunnan Agricultural University, Kunming 650201, China; lanyiquan2025@163.com (Y.L.); 18387637540@163.com (L.H.); 4 College of Information Engineering, College of Arts and Sciences Kunming, Kunming 650222, China; kmykg163@163.com

**Keywords:** xanthoxylin, alcoholic liver injury, molecular dynamics simulation, EGFR/AKT pathway, molecular mechanisms

## Abstract

Alcoholic liver injury (ALI) represents a significant global health burden with limited therapeutic options. Xanthoxylin, a natural flavonoid compound, has demonstrated potential hepatoprotective properties, yet its underlying molecular mechanisms against ALI remain poorly elucidated. This study employed an integrated strategy combining network pharmacology, molecular docking, molecular dynamics (MD) simulations, and in vitro experimental validation to systematically investigate the protective mechanisms of xanthoxylin against ALI. Network pharmacology screening identified 52 intersection targets between xanthoxylin and ALI, with the top 10 core targets comprising ALB, PPARG, BCL2, PTGS2, ESR1, HIF1A, EGFR, HSP90AA1, GSK3B, and PARP1. GO enrichment analysis highlighted mitochondrion and mitochondrial outer membrane among the top 10 cellular component (CC) terms. KEGG pathway analysis revealed PI3K-Akt signaling within the top 10 pathways. Molecular docking suggested potential binding of xanthoxylin to the key targets. Subsequent MD simulations further confirmed the formation of stable complexes between xanthoxylin and EGFR, PPARG, and PTGS2. In vitro, xanthoxylin significantly ameliorated ethanol-induced HepG2 cell injury, attenuated TC and TG elevations, suppressed mitochondrial ROS accumulation, and enhanced SOD activity. Mechanistically, xanthoxylin upregulated HSP90, p-EGFR, EGFR, p-AKT, AKT, and PPARG protein expression, and suppressed the expression levels of PTGS2. Erlotinib, an EGFR inhibitor, reversed the cytoprotective effects of xanthoxylin. Xanthoxylin protects against alcoholic liver injury through regulating the EGFR/AKT pathway, with concurrent modulation of PPARG and PTGS2. These findings provide compelling evidence for xanthoxylin as a promising therapeutic candidate for ALI and establish a foundation for subsequent preclinical development.

## 1. Introduction

Alcoholic liver disease (ALD) represents a major global health burden, encompassing a spectrum of hepatic pathologies ranging from simple steatosis to alcoholic hepatitis, fibrosis, and ultimately cirrhosis and hepatocellular carcinoma [1]. Chronic and excessive alcohol consumption triggers a cascade of deleterious events in hepatocytes, including oxidative stress, lipid accumulation, inflammatory responses, and mitochondrial dysfunction, which collectively contribute to hepatocellular injury and death [2,3]. Despite the significant morbidity and mortality associated with ALD, therapeutic options remain limited, with abstinence being the cornerstone of treatment. However, relapse is common, and pharmacological interventions such as corticosteroids for severe alcoholic hepatitis exhibit only modest efficacy and considerable side effects [4]. Therefore, the discovery of novel therapeutic agents with hepatoprotective properties and well-defined molecular mechanisms is urgently needed.

Natural products have historically served as invaluable sources for drug discovery, offering structural diversity and favorable safety profiles that render them attractive candidates for the development of ALD therapeutics [5]. Xanthoxylin, a natural flavonoid isolated from *Zanthoxylum* species, has attracted increasing scientific attention due to its diverse pharmacological activities, including anti-inflammatory, antioxidant, antimicrobial, and anticancer effects [6]. Previous studies have demonstrated that xanthoxylin exerts protective effects against various cellular stressors by modulating oxidative stress and inflammatory signaling pathways [7,8]. Nevertheless, the potential hepatoprotective efficacy of xanthoxylin against alcohol-induced liver injury and its underlying molecular mechanisms have not been systematically investigated.

The traditional drug discovery paradigm often follows a linear “one-target, one-drug” approach, which may be insufficient for capturing the complex, multifactorial nature of ALD pathogenesis. In recent years, network pharmacology has emerged as a powerful systems-level methodology that integrates polypharmacology, systems biology, and bioinformatics to elucidate the multi-target, multi-pathway mechanisms of bioactive compounds [9,10]. By constructing and analyzing biological networks comprising drug-target, protein–protein, and pathway interactions, network pharmacology enables the identification of key therapeutic targets and signaling pathways from a holistic perspective, thereby providing a rational framework for mechanism-based drug discovery [11]. Complementarily, molecular docking and molecular dynamics (MD) simulations offer atomistic insights into the binding modes, affinities, and conformational dynamics of ligand–protein interactions, facilitating the prioritization of candidate targets for experimental validation [12,13].

The phosphoinositide 3-kinase/protein kinase B (PI3K/Akt) signaling pathway constitutes a critical node in cellular homeostasis, governing fundamental biological processes including cell survival, proliferation, metabolism, and stress response [14]. In the context of ALD, emerging evidence indicates that ethanol exposure suppresses PI3K/Akt pathway activity, thereby exacerbating hepatocyte apoptosis, steatosis, and oxidative damage [15,16]. Conversely, pharmacological activation of the PI3K/Akt cascade has been shown to confer significant protection against alcohol-induced hepatocellular injury [17]. Epidermal growth factor receptor (EGFR), a receptor tyrosine kinase that serves as a key upstream activator of the PI3K/Akt pathway, plays pivotal roles in hepatocyte regeneration and cytoprotection [18]. Notably, peroxisome proliferator-activated receptor gamma (PPARG) and prostaglandin-endoperoxide synthase 2 (PTGS2), as downstream or associated mediators of PI3K/Akt signaling, are intimately involved in the regulation of antioxidant defenses, hepatic lipid metabolism and inflammatory responses [19,20]. However, whether xanthoxylin modulates the EGFR-mediated PI3K/Akt signaling to ameliorate ALD remains entirely unknown.

In the present study, we employed an integrated strategy combining network pharmacology, molecular docking, MD simulations, and cellular experiments to systematically investigate the protective effects of xanthoxylin against ethanol-induced hepatocyte injury and to elucidate its underlying molecular mechanisms. Our findings reveal that xanthoxylin protects against alcoholic liver injury by regulating the EGFR/Akt pathway, thereby restoring PPARG and PTGS2 expression, attenuating mitochondrial reactive oxygen species (ROS) production, enhancing SOD activity, and ameliorating lipid accumulation. These results provide novel mechanistic insights into the hepatoprotective action of xanthoxylin and support its potential as a promising candidate for ALD intervention.

## 2. Results

### 2.1. Construction of Intersection Target Network Between Xanthoxylin and Alcoholic Liver Injury

**Figure 1:**
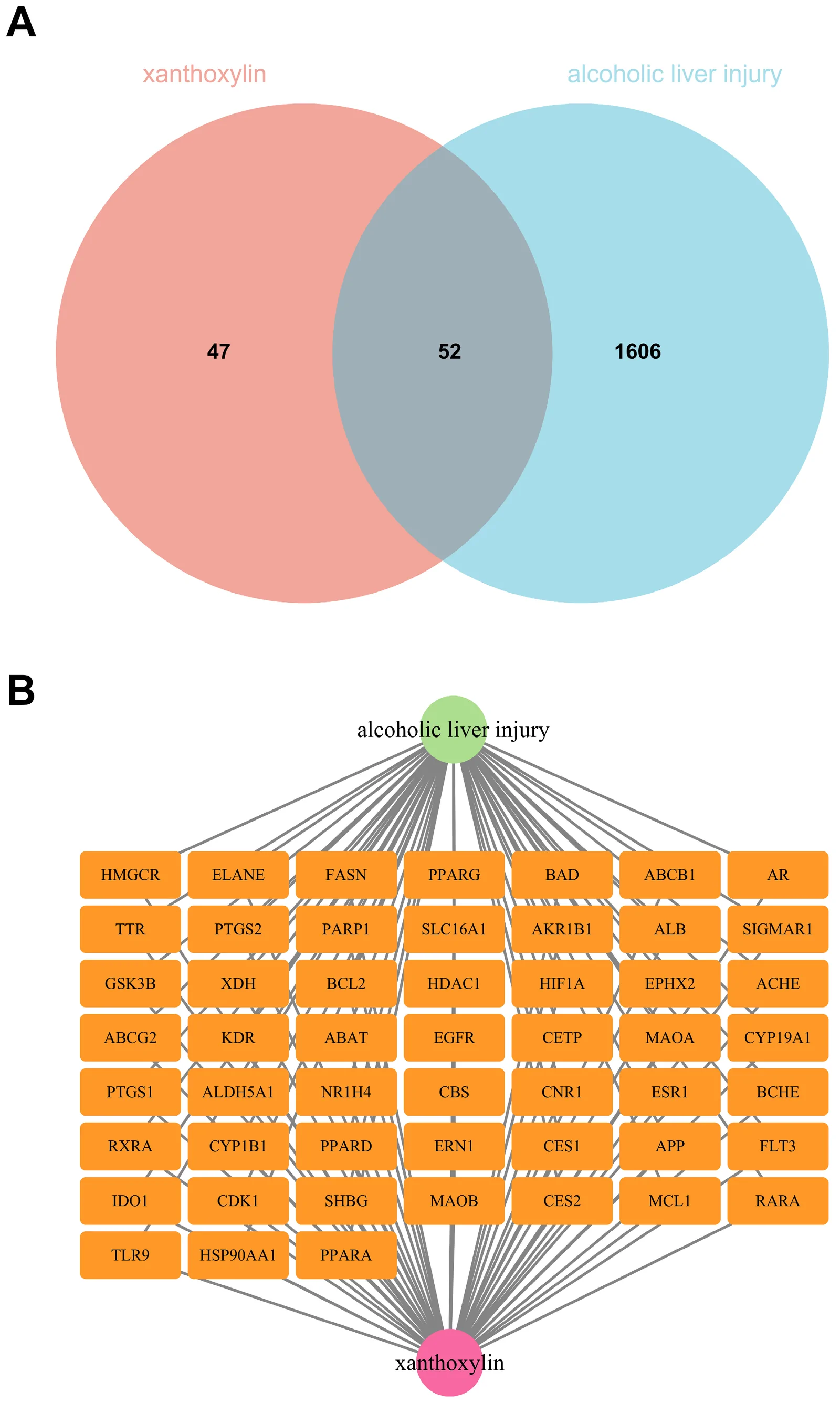
Intersected gene targets between xanthoxylin and alcoholic liver injury are visualized. (**A**) The intersection of gene targets between xanthoxylin and alcoholic liver injury was exhibited by a Venn diagram. (**B**) Visualization of intersected gene targets between xanthoxylin and alcoholic liver injury via Cytoscape software. The orange nodes represent the intersected gene targets between xanthoxylin and alcoholic liver injury.

Using SwissTargetPrediction, a total of 99 potential targets of xanthoxylin with probability scores greater than 0 were identified. Meanwhile, 1658 target genes associated with alcoholic liver injury were retrieved from the GeneCards database. The overlapping targets between xanthoxylin and alcoholic liver injury were determined using the Sangerbox online tool, which revealed 52 intersecting targets with potential therapeutic value against alcoholic liver injury (Figure 1A). Subsequently, a “xanthoxylin–targets–alcoholic liver injury” network was constructed using Cytoscape 3.10.4, in which the orange nodes represent the shared targets between xanthoxylin and the disease (Figure 1B).

### 2.2. Analysis of PPI Network of Targets Between Xanthoxylin and Alcoholic Liver Injury

**Figure 2:**
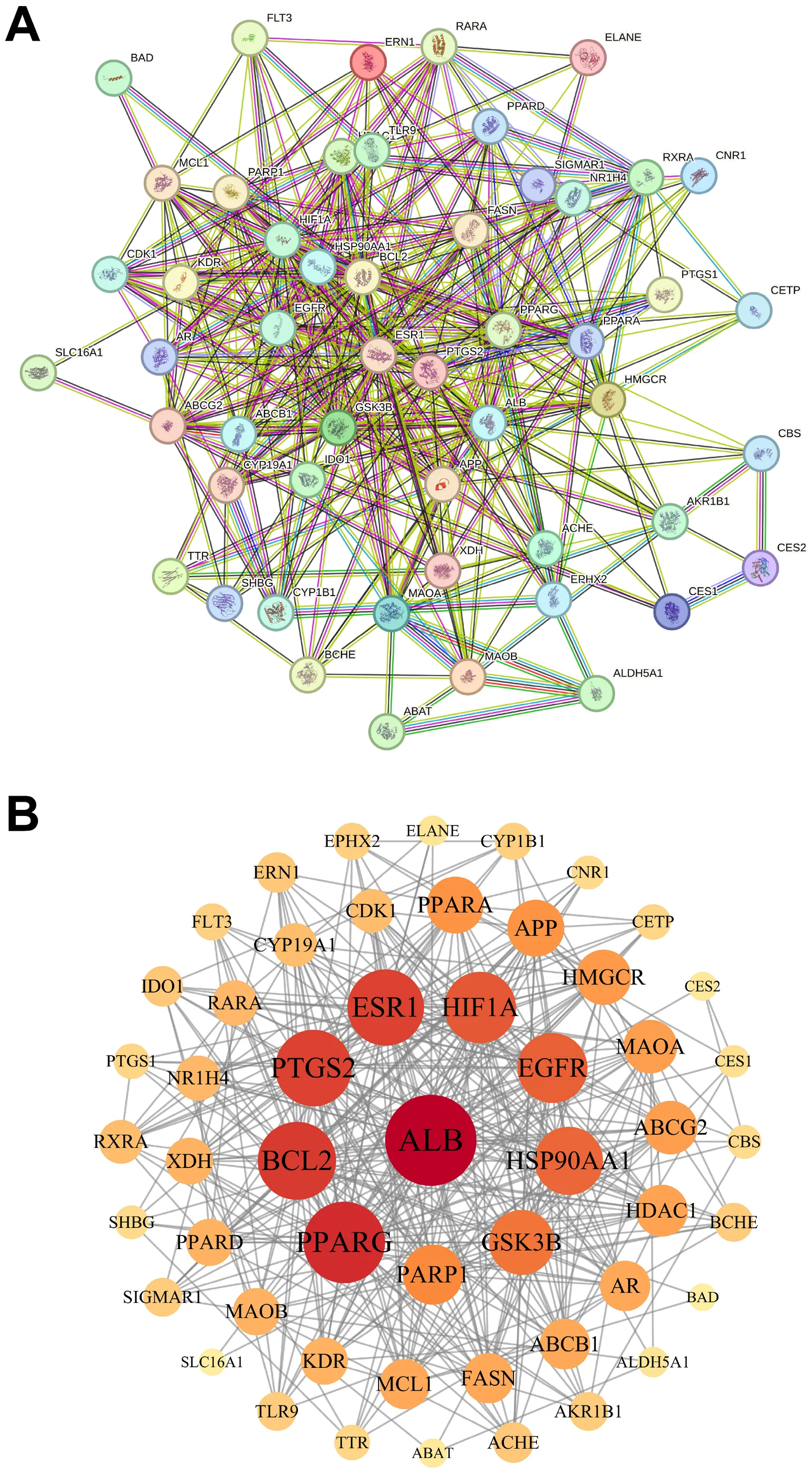
Identification of key targets of xanthoxylin against alcoholic liver injury. (**A**) The PPI network diagram was constructed using the STRING database. (**B**) The core targets were identified by using Cytoscape software. Larger and darker red nodes represent more important targets in the network.

A protein–protein interaction (PPI) network for the 52 candidate targets of xanthoxylin implicated in alleviating alcoholic liver injury was constructed using the STRING database and visualized with Cytoscape 3.10.4. The PPI network contained 52 nodes and 375 edges, with an average node degree of 14.4 (Figure 2A). As shown in Figure 2B, the size and color of each node correspond to the interaction strength, where larger and darker nodes represent stronger protein–protein associations, implying more complex and dense connectivity among the proteins. According to degree ranking, the top 10 hub targets were identified as ALB, PPARG, BCL2, PTGS2, ESR1, HIF1A, EGFR, HSP90AA1, GSK3B, and PARP1.

### 2.3. GO and KEGG Enrichment Analysis of Potential Targets of Xanthoxylin in Alleviating Alcoholic Liver Injury

**Figure 3:**
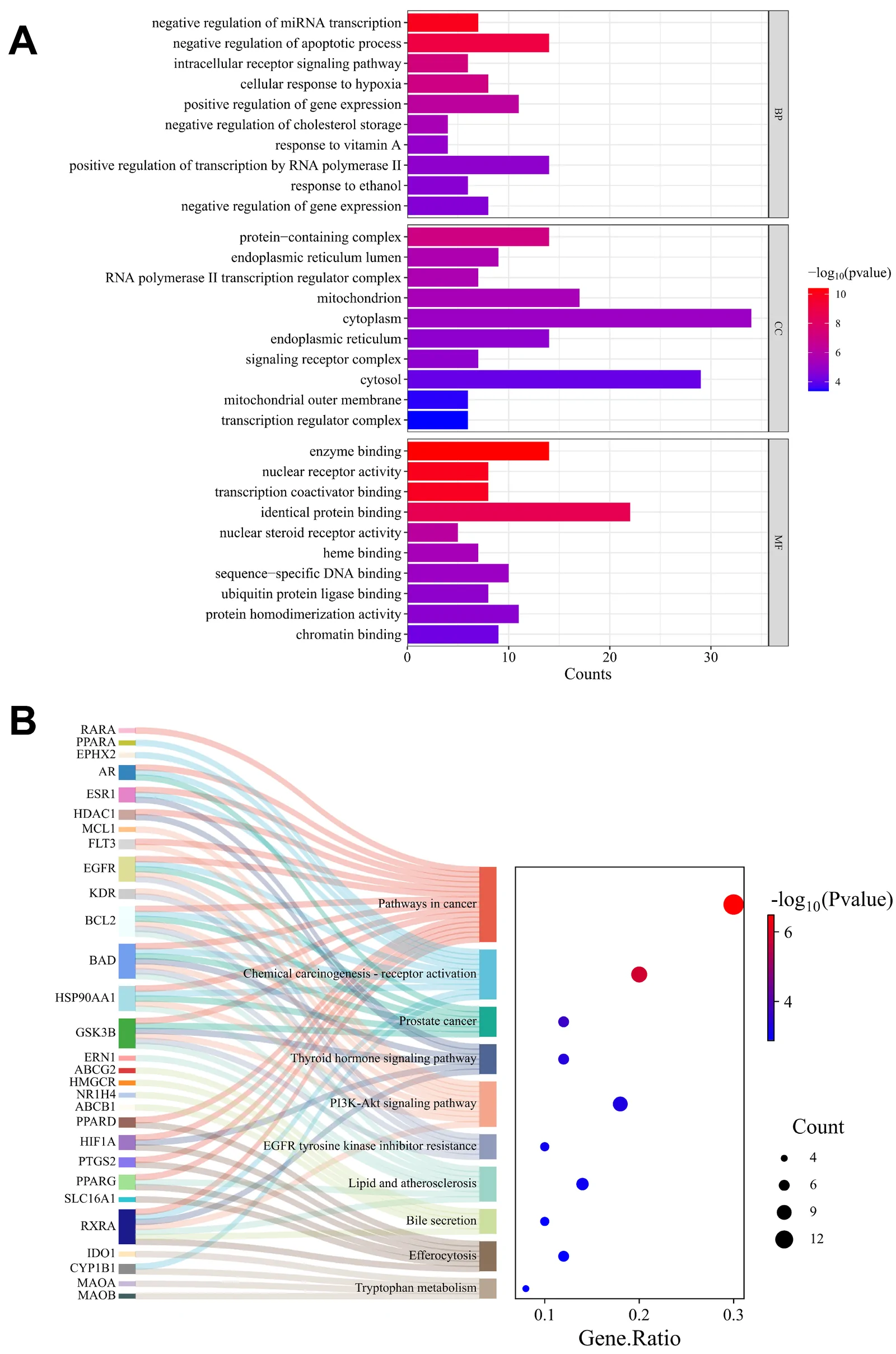
GO and KEGG enrichment analyses for the mechanisms of xanthoxylin in alleviating alcoholic liver injury. (**A**) GO enrichment analysis indicates the top 10 of BP, CC, and MF terms at *p* < 0.01. (**B**) KEGG enrichment analysis indicates the top 10 KEGG terms at *p* < 0.01.

To further explore the potential mechanisms underlying the protective effects of xanthoxylin against alcohol-induced liver injury, GO and KEGG enrichment analyses were performed based on UniProt annotations, with *p* < 0.01 considered statistically significant. GO enrichment analysis identified 77 biological process (BP), 18 cellular component (CC), and 38 molecular function (MF) terms (Figure 3A). The major BP terms included negative regulation of miRNA transcription, negative regulation of apoptotic processes, intracellular receptor signaling, cellular response to hypoxia, positive regulation of gene expression, negative regulation of cholesterol storage, response to vitamin A, positive regulation of transcription by RNA polymerase II, response to ethanol, and negative regulation of gene expression. The principal CC terms included protein-containing complexes, endoplasmic reticulum lumen, RNA polymerase II transcription regulator complexes, mitochondria, cytoplasm, endoplasmic reticulum, signaling receptor complexes, cytosol, mitochondrial outer membrane, and transcription regulator complexes. The predominant MF terms were enzyme binding, nuclear receptor activity, transcription coactivator binding, identical protein binding, nuclear steroid receptor activity, heme binding, sequence-specific DNA binding, ubiquitin protein ligase binding, protein homodimerization activity, and chromatin binding. KEGG enrichment analysis identified 20 significantly enriched pathways. The top 10 pathways included pathways in cancer, chemical carcinogenesis–receptor activation, prostate cancer, thyroid hormone signaling, PI3K–Akt signaling, EGFR tyrosine kinase inhibitor resistance, lipid and atherosclerosis, bile secretion, efferocytosis, and tryptophan metabolism (Figure 3B). Notably, ALB and PARP1 were not mapped to any of the top 10 enriched KEGG pathways. Furthermore, the enrichment of the PI3K–Akt signaling pathway indicates that this pathway may be associated with the protective effects of xanthoxylin against alcohol-induced liver injury.

### 2.4. Molecular Docking of Xanthoxylin to Key Targets

**Figure 4:**
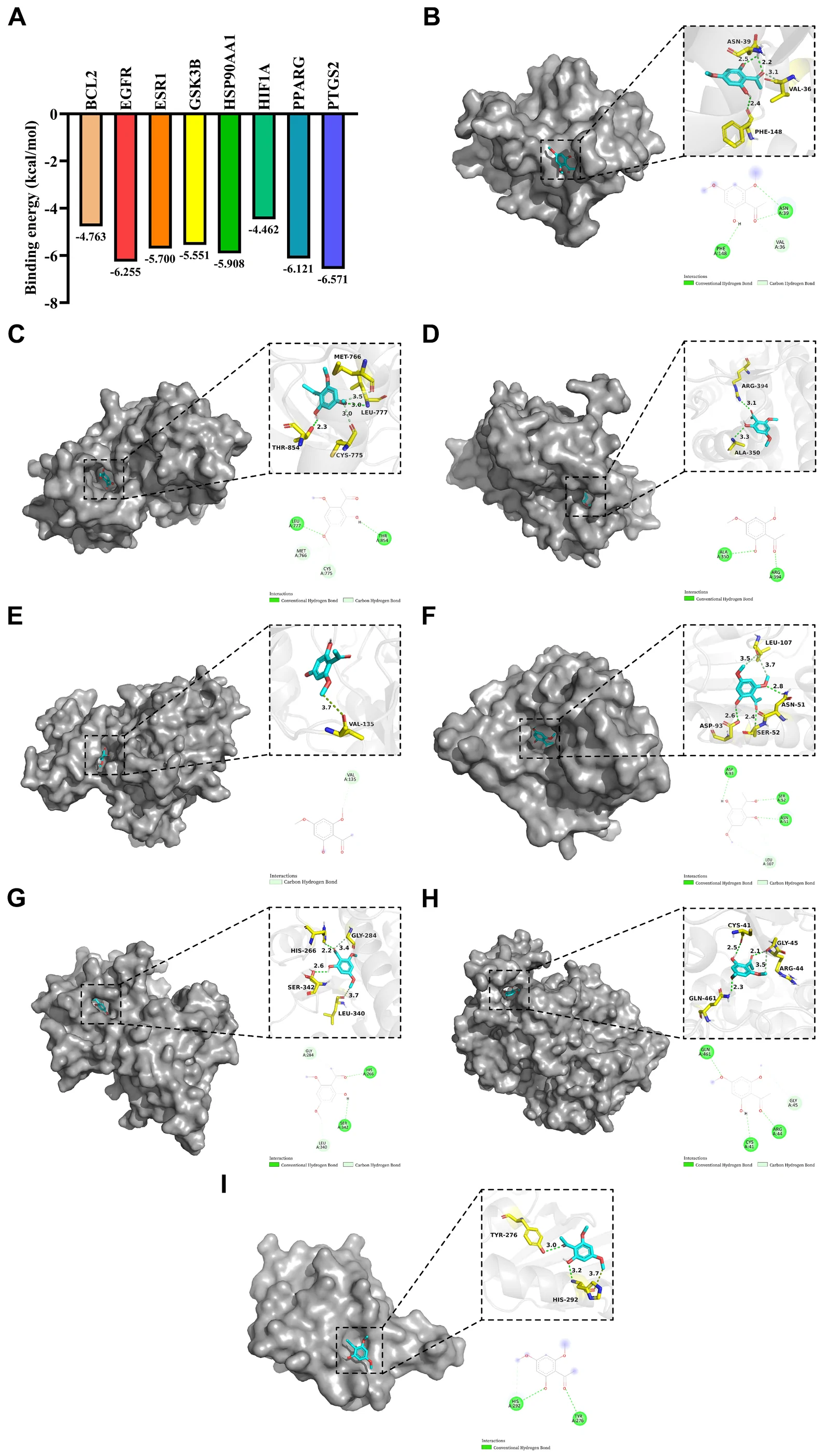
Molecular docking of xanthoxylin with its potential key targets. (**A**) Molecular docking results of xanthoxylin with the potential key targets. (**B**–**I**) Docking modes of xanthoxylin with BCL2 (**B**), EGFR (**C**), ESR1 (**D**), GSK3B (**E**), HSP90AA1 (**F**), PPARG (**G**), PTGS2 (**H**), and HIF1A (**I**), respectively.

Based on the results of KEGG enrichment, we selected the eight key target proteins to evaluate the binding energy and models of xanthoxylin-receptors by using molecular docking, including EGFR, HSP90AA1, GSK3B, BCl2, PPARG, PTGS2, ESR1, and HIF1A. As shown in Figure 4A, the docking results showed that all target proteins exhibited favorable binding affinities toward xanthoxylin, except for BCL2, whose score was higher than –5 kcal/mol. Among these targets, PTGS2, EGFR, and PPARG displayed their binding energy with scores all lower than −6 kcal/mol. Additionally, the binding energies of ESR1 and HSP90AA1 were −5.312 kcal/mol and −5.908 kcal/mol, respectively.

Subsequently, we analyzed the 3D and 2D binding models of xanthoxylin with the key targets, as shown in Figure 4B–I. Xanthoxylin bound to the binding pockets of BCL2, EGFR, ESR1, GSK3B, HSP90AA1, PPARG, PTGS2, and HIF1A, and formed hydrogen bonds with these proteins to stabilize the binding complexes. Specifically, xanthoxylin formed hydrogen bonds with ASN-39, PHE-148, and VAL-36 in BCL2; with THR-854, LEU-777, CYS-775, and MET-766 in EGFR; with PHE-119 and ASP-126 in ESR1; with VAL-135 and GLN-185 in GSK3B; with ASP-93, SER-52, ASN-51, and LEU-107 in HSP90AA1; with HIS-266, SER-342, GLY-284, and LEU-340 in PPARG; and with GLY-45, GLN-461, CYS-41, and ARG-44 in PTGS2; and with TYR-276 and HIS-292 in HIF1A. Collectively, these findings suggested that xanthoxylin might interact with the key targets associated with alcoholic liver injury, thereby further verifying the results of the network pharmacology analysis.

### 2.5. Verification of MD Simulations

**Figure 5:**
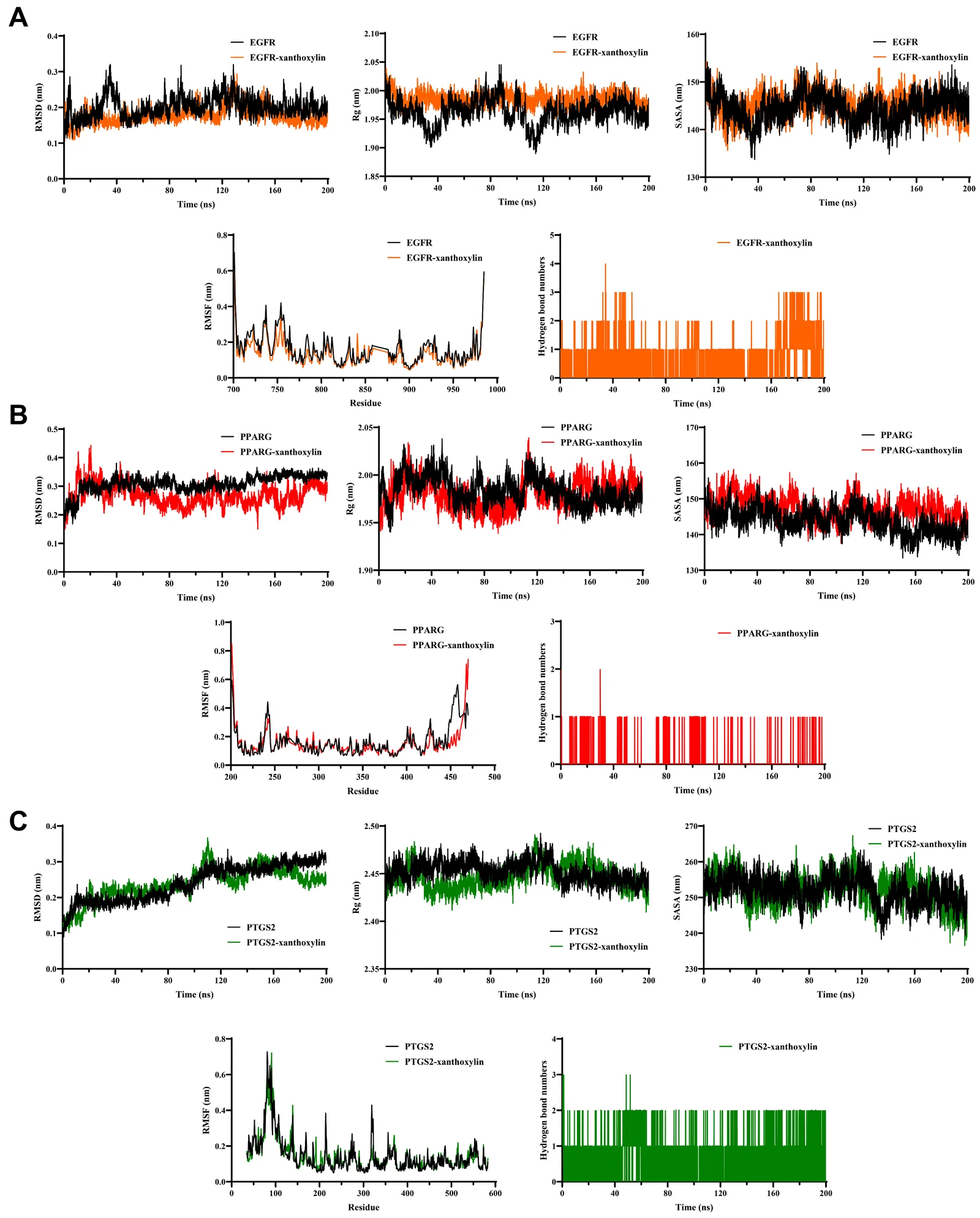
Molecular dynamics simulations of the interactions of xanthoxylin with EGFR, PPARG and PTGS2 for 200 ns. (**A**) The RMSD, Rg, SASA, RMSF, and hydrogen bond numbers of free EGFR and the EGFR–xanthoxylin complex. (**B**) The RMSD, Rg, SASA, RMSF and hydrogen bond numbers of free PPARG and the PPARG–xanthoxylin complex. (**C**) The RMSD, Rg, SASA, RMSF, and hydrogen bond numbers of free PTGS2 and the PTGS2-xanthoxylin complex.

To further verify the binding stability of protein–ligand complexes, the three systems (EGFR–xanthoxylin, PPARG–xanthoxylin, and PTGS2–xanthoxylin) were selected as representatives to conduct 200 ns molecular dynamics (MD) simulations. Key MD parameters, including root-mean-square deviation (RMSD), radius of gyration (Rg), root-mean-square fluctuation (RMSF), solvent-accessible surface area (SASA), and hydrogen bond numbers, were systematically analyzed (Figure 5A–C).

RMSD was used to assess system equilibration. The RMSD trajectories of the three ligand-bound complexes were generally comparable to those of their respective free proteins, except that the xanthoxylin–EGFR complex exhibited a notably lower average RMSD (0.1754 ± 0.0225 nm) than free EGFR (0.2000 ± 0.0295 nm). Rg and SASA analyses, which respectively monitor protein compactness and solvent exposure at the binding interface, revealed nearly identical profiles between each complex and its apo counterpart, indicating that xanthoxylin induced only minor perturbations to the global conformation of the target proteins. RMSF analysis quantified residue-specific flexibility. Binding of xanthoxylin to PPARG significantly reduced the fluctuations of residues 447–461, suggesting a stabilizing effect on the AF-2 functional region crucial for PPARG activity. In contrast, the RMSF profiles of the EGFR and PTGS2 complexes remained largely unchanged relative to their apo forms, implying that ligand binding did not appreciably affect the intrinsic flexibility of these two proteins. The number of intermolecular hydrogen bonds formed between xanthoxylin and each target was monitored over the simulation. The counts fluctuated within the ranges of 0–4 (EGFR), 0–2 (PPARG), and 0–3 (PTGS2), respectively.

Collectively, the systematic evaluation of the five MD parameters demonstrates that xanthoxylin can form stable complexes with the three targets, thereby corroborating the reliability of the simulation results.

### 2.6. The Effect of Xanthoxylin in Ameliorating Alcoholic Liver Injury in HepG2 Cells

**Figure 6:**
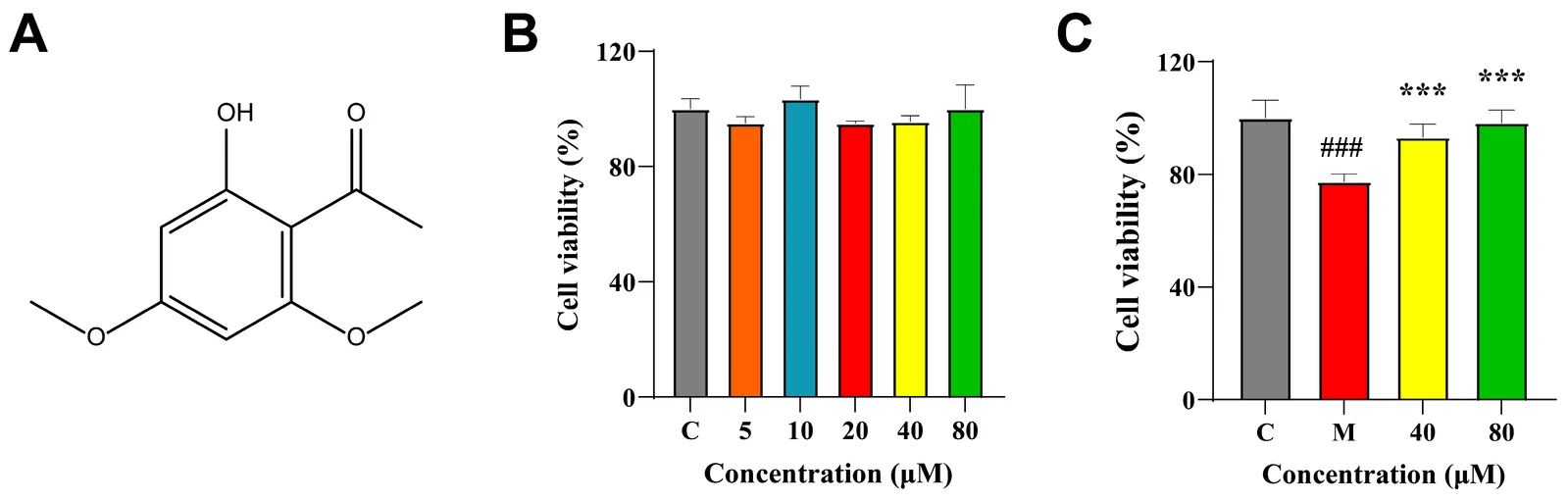
The effect of xanthoxylin on the survival rate of HepG2 cells. (**A**) The chemical structure of xanthoxylin. (**B**) The effect of different concentrations of xanthoxylin on HepG2 cell viability. (**C**) The effect of different concentrations of xanthoxylin on cell viability in ethanol-induced HepG2 cell injury. Data are presented as the mean ± SEM (*n* = 3). ### *p* < 0.001 vs. the control; *** *p* < 0.001 vs. the model.

The chemical structure of xanthoxylin is presented in Figure 6A. To evaluate its potential cytotoxicity, we performed MTT assays on HepG2 cells treated with various concentrations of the compound. As shown in Figure 6B, xanthoxylin exhibited no significant cytotoxic effects on HepG2 cells within the concentration range of 0 to 80 μM. Therefore, concentrations of 40 μM and 80 μM were selected for subsequent experiments. Based on our previous study [12], 400 mM of ethanol was selected to induce the cell model. As shown in Figure 6C, the results demonstrated that ethanol stimulation led to a marked decrease in cell viability in the model group. However, treatment with xanthoxylin significantly alleviated ethanol-induced cellular damage in a dose-dependent manner, with a more pronounced protective effect observed at the higher concentration.

**Figure 7:**
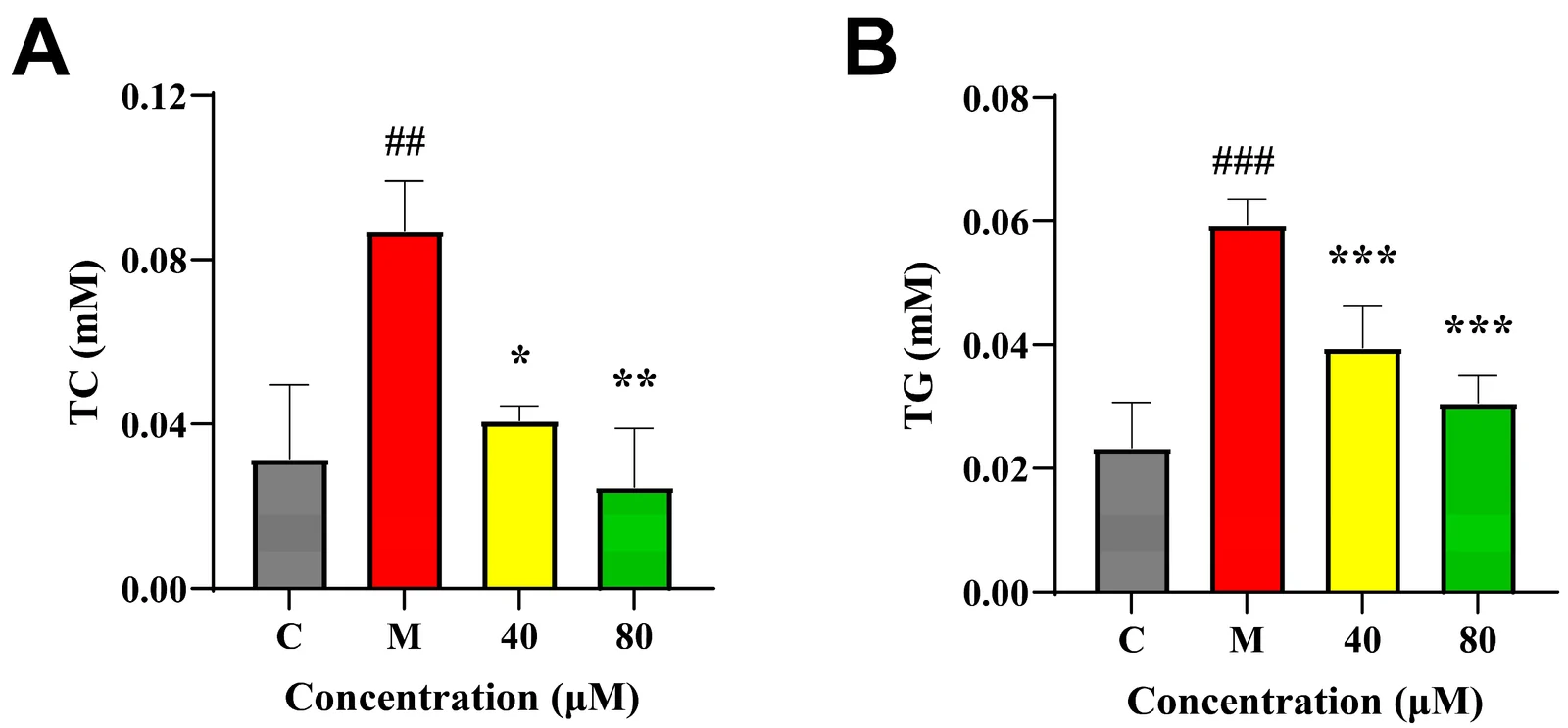
Xanthoxylin reduced the levels of TC and TG in ethanol-induced HepG2 cells. (**A**) Xanthoxylin reduced the content of TC in HepG2 cells. (**B**) Xanthoxylin reduced the content of TG in HepG2 cells. Data are presented as the mean ± SEM (*n* = 3). ## *p* < 0.01, ### *p* < 0.001 vs. the control; * *p* < 0.05, ** *p* < 0.01, *** *p* < 0.001 vs. the model.

Hepatic levels of total cholesterol (TC) and triglycerides (TG) are key indicators of lipid accumulation, and their elevation is closely linked to hepatic dysfunction [21]. To further evaluate the preventive role of xanthoxylin against alcoholic liver injury, we measured intracellular TC and TG levels (Figure 7A,B). Compared with the model group, treatment with 40 μM and 80 μM xanthoxylin significantly reduced the TC and TG contents in HepG2 cells, thereby effectively restoring normal lipid metabolism. Collectively with our previous observations, these results further corroborate the hepatoprotective effect of xanthoxylin.

**Figure 8:**
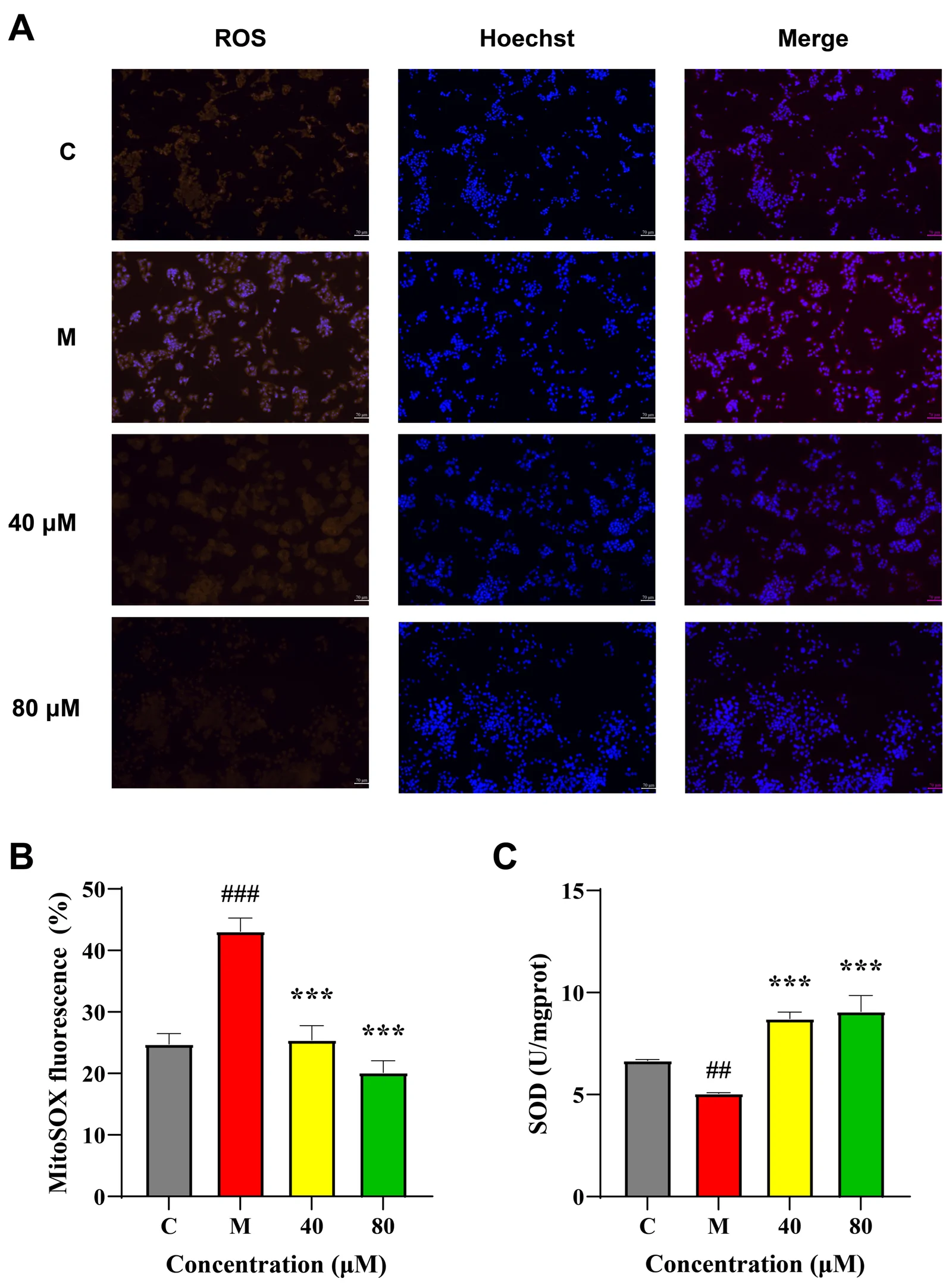
Xanthoxylin reduces the levels of mitochondrial ROS and enhances SOD activity in HepG2 cells. (**A**) The levels of ROS in cell mitochondria treated by different doses of xanthoxylin. (**B**) Quantitative analysis of ROS levels in cell mitochondria. (**C**) The levels of SOD activity was measured. Data are presented as the mean ± SEM (*n* = 3). ## *p* < 0.01, ### *p* < 0.001 vs. the control; *** *p* < 0.001 vs. the model.

Ethanol and its metabolites are known to trigger excessive reactive oxygen species (ROS) generation in hepatocytes, disrupting redox homeostasis and inducing oxidative stress and lipid peroxidation, which ultimately lead to cellular damage [21]. Given that mitochondria are a major source of ethanol-induced ROS, we measured mitochondrial ROS levels in ethanol-stimulated HepG2 cells using fluorescence microscopy and quantified the fluorescence intensity with ImageJ 1.52 software (Figure 8A,B). As illustrated, ethanol exposure caused a marked increase in mitochondrial ROS compared with the control group. Notably, xanthoxylin treatment significantly alleviated this ROS accumulation, with a more pronounced scavenging effect observed at the higher concentration (80 μM). Furthermore, xanthoxylin significantly enhanced SOD activity (Figure 8C).

### 2.7. Verification of Key Targets Related to Xanthoxylin in Ameliorating Alcoholic Liver Injury

**Figure 9:**
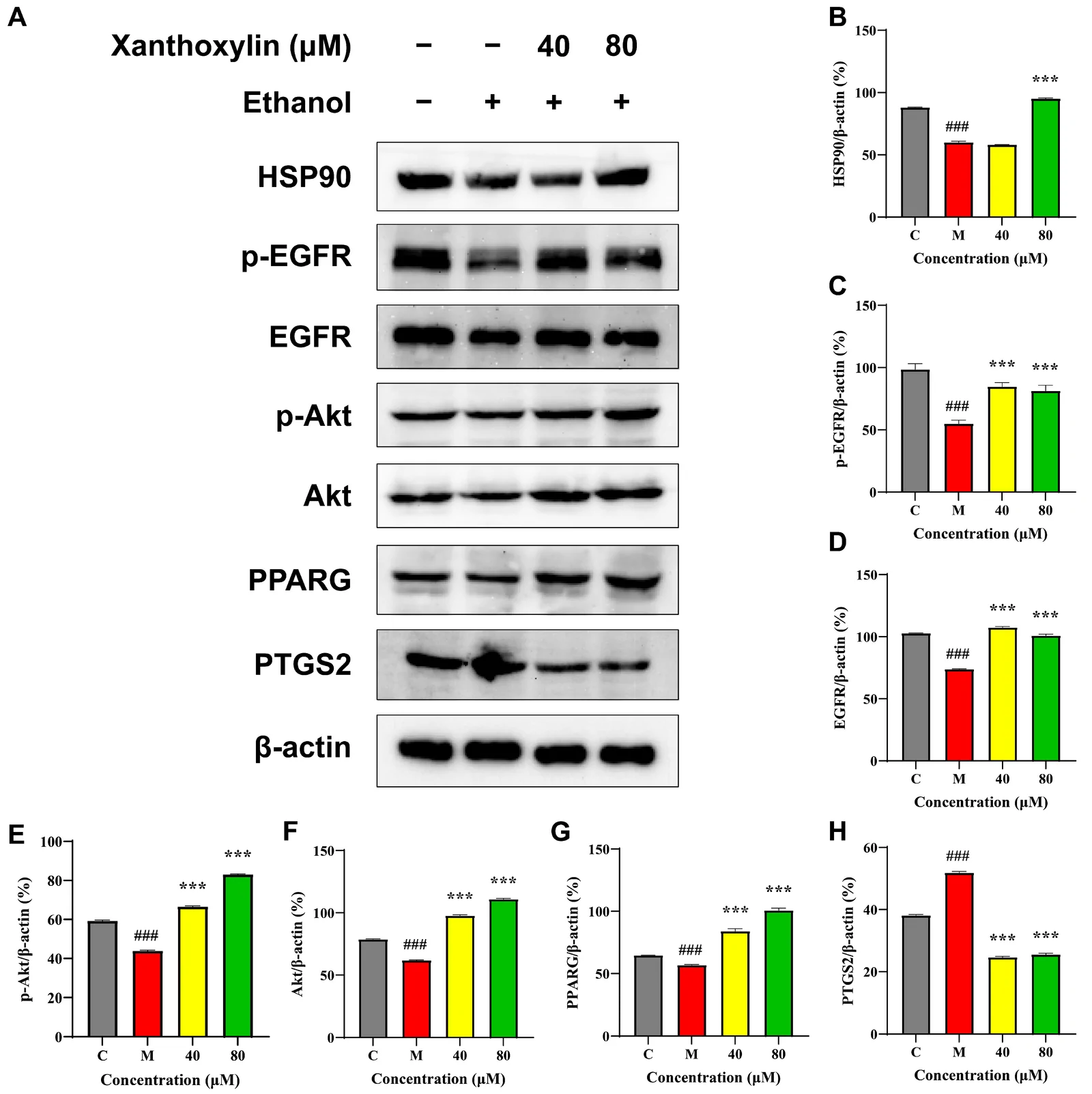
The effect of xanthoxylin in the amelioration of alcoholic liver injury in HepG2 cells. (**A**) Western blotting was conducted to detect the effect of xanthoxylin on the expression of the key target proteins. (**B**–**H**) Quantification of the expression levels of HSP90 (**B**), p-EGFR (**C**), EGFR (**D**), p-Akt (**E**), Akt (**F**), PPARG (**G**), and PTGS2 (**H**). Data are presented as the mean ± SEM (*n* = 3). ### *p* < 0.001 vs. the control; *** *p* < 0.001 vs. the model.

Based on the results of network pharmacology, molecular docking, and MD simulations, to further confirm whether the anti-alcoholic liver injury effects of xanthoxylin are related to the key target, we conducted a Western blotting assay to detect the effect of xanthoxylin on the expression levels of key target proteins, including p-RGFR, EGFR, p-Akt, Akt, HSP90, PTGS2, and PPARG. As shown in Figure 9A–H, xanthoxylin at 40 μM and 80 μM significantly promoted the protein expression levels of p-EGFR, EGFR, p-Akt, Akt, HSP90, and PPARG, while inhibiting the expression levels of PTGS2. These results indicated that the anti-alcoholic liver injury efficacy of xanthoxylin is associated with the regulation of these key targets.

**Figure 10:**
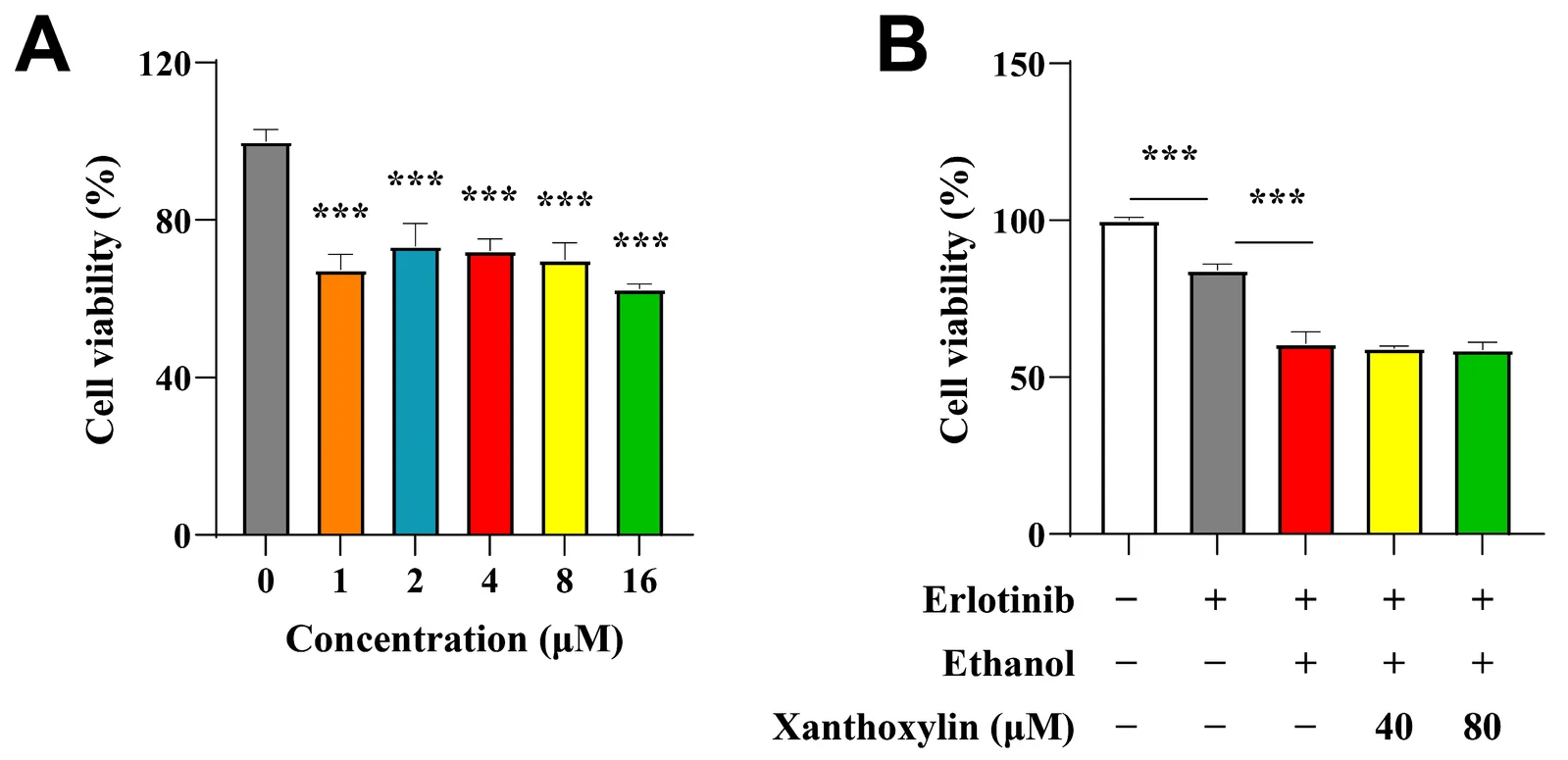
Erlotinib reversed the anti-alcoholic liver injury effects of xanthoxylin. (**A**) The effect of different concentrations of erlotinib on HepG2 cell viability. (**B**) Effects of varying concentrations of xanthoxylin on ethanol-induced HepG2 cell injury in the presence of erlotinib. Data are presented as the mean ± SEM (*n* = 3). *** *p* < 0.001 vs. the control.

To further investigate the role of the EGFR signaling pathway in the efficacy of xanthoxylin against alcoholic liver injury, erlotinib, an EGFR kinase inhibitor, was used to test whether the effects of xanthoxylin were dependent on this pathway. As shown in Figure 10A, 1–16 μM erlotinib significantly inhibited the proliferation of HepG2 cells. Subsequently, 1 μM erlotinib was selected for further experiments. As shown in Figure 10B, when cells were treated with erlotinib, the protective effects of xanthoxylin against ethanol-induced injury were no longer observed. These results suggested that xanthoxylin may alleviate ethanol-induced damage in HepG2 cells via EGFR signaling.

## 3. Discussion

Alcoholic liver disease (ALD) encompasses a spectrum of hepatic pathologies ranging from steatosis to steatohepatitis, fibrosis, and cirrhosis, with limited FDA-approved pharmacotherapies available [2]. Natural products have garnered substantial attention as alternative therapeutic strategies due to their multi-target regulatory capabilities and favorable safety profiles [5]. Xanthoxylin is a bioactive flavonoid derived from *Zanthoxylum* species, which is officially listed in China’s catalog of medicinal and edible substances. This ingredient exhibits diverse pharmacological activities including anti-inflammatory, antioxidant, and anti-tumor effects [7,8,22,23]. However, its hepatoprotective potential against ALI and the underlying molecular mechanisms have not been systematically investigated. The present study integrates computational and experimental approaches to elucidate how xanthoxylin ameliorates ALI primarily through the EGFR/AKT pathway, complemented by PPARG and PTGS2 modulation.

The network pharmacology approach has emerged as a powerful paradigm for deciphering the polypharmacological characteristics of traditional medicines [10]. Our screening identified 52 shared targets between xanthoxylin and ALI, with PPI network topology analysis revealing ALB, PPARG, BCL2, PTGS2, ESR1, HIF1A, EGFR, HSP90AA1, GSK3B, and PARP1 as the top 10 hub genes. Notably, the enrichment of mitochondrion and mitochondrial outer membrane within GO cellular component terms aligns with the established central role of mitochondrial dysfunction in ALI pathogenesis [24]. Ethanol metabolism disrupts mitochondrial electron transport chain efficiency, precipitating excessive mitochondrial ROS generation, lipid peroxidation, and hepatocellular apoptosis [25]. The observed xanthoxylin-mediated suppression of mitochondrial ROS in our study substantiates the computational prediction of mitochondrial involvement.

The PI3K-AKT pathway emerged as a significantly enriched KEGG pathway, consistent with its established roles in hepatocyte survival and liver regeneration [26]. Dysregulation of this pathway has been implicated in alcohol-related alterations of hepatocyte survival, metabolism, and autophagy. The exclusion of ALB and PARP1 from pathway enrichment, despite their high connectivity in the PPI network, suggests their potential role as general physiological modulators rather than pathway-specific signaling nodes in this context.

Molecular docking and MD simulations provided atomic-level insights into xanthoxylin–target interactions. The 200 ns MD trajectories demonstrated stable complex formation for EGFR, PPARG, and PTGS2, with particularly intriguing dynamics for EGFR–xanthoxylin. The ligand-bound EGFR exhibited superior conformational stability compared to its apo form, as manifested by diminished RMSD fluctuations, reduced radius of gyration (Rg), and sustained hydrogen bond networks. This “stabilizing binding” phenomenon, where small molecule engagement enhances rather than compromises protein structural integrity, has been increasingly recognized in drug–target interaction studies [27]. For EGFR specifically, this stabilization may reflect xanthoxylin’s capacity to lock the kinase domain in a favorable conformation.

The in vitro validation substantiated our computational predictions. Ethanol exposure at 400 mmol/L induced significant HepG2 cell injury, recapitulating key features of ALI including dyslipidemia and oxidative stress [28,29]. Xanthoxylin intervention at 40 and 80 μM concentrations dose-dependently restored cell viability, normalized TC and TG levels, enhanced SOD activity, and attenuated mitochondrial ROS accumulation. These functional improvements correlated with Western blot-detected alterations in HSP90, p-EGFR, EGFR, p-AKT, AKT, PPARG, and PTGS2 protein expression.

EGFR activation represents another finding warranting detailed discussion. In our study, ethanol significantly reduced p-EGFR, total EGFR, p-AKT, and total AKT protein levels in HepG2 cells, while xanthoxylin treatment effectively reversed this suppression. Although early studies suggested that ethanol impairs EGFR function primarily through inhibition of autophosphorylation without altering total receptor abundance [30], subsequent evidence from ethanol-fed animals and alcoholic hepatitis patients has demonstrated a downregulation of EGFR protein [31,32], supporting our observation. The restoration of total EGFR by xanthoxylin may be partially mediated via HSP90 upregulation, given that HSP90 stabilizes EGFR in a signaling-competent conformation [33,34]. Meanwhile, the recovery of total AKT complements the enhanced phosphorylation, collectively indicating that xanthoxylin protects against ethanol-induced hepatocyte injury through both quantitative restoration and functional activation of the EGFR/AKT signaling pathway.

In the present study, ethanol exposure caused HepG2 cell injury, intracellular TC and TG accumulation, mitochondrial ROS elevation, and downregulation of PPARG, whereas treatment with the compound restored PPARG expression and suppressed PTGS2 expression. These effects were accompanied by reduced intracellular lipid accumulation, decreased mitochondrial ROS, and improved cell viability, suggesting that the compound may protect ethanol-exposed HepG2 cells through coordinated regulation of metabolic, inflammatory, and redox homeostasis. In this model, PPARG may function primarily as a metabolic-antioxidant regulator. Restoration of PPARG signaling could enhance hepatocellular antioxidant defenses and improve lipid metabolic homeostasis, which may contribute to the reduction in mitochondrial oxidative stress and intracellular TC and TG levels. This interpretation is supported by a previous HepG2 study showing that PPARG participated in the regulation of SOD expression during protection against ethanol-induced oxidative stress [35]. PTGS2 may represent a complementary inflammatory lipid-mediator pathway. Ethanol-induced COX-2/PGE_2_ signaling has been associated with alcohol-related hepatic lipid accumulation, whereas suppression of PTGS2 may attenuate prostaglandin-mediated inflammatory amplification and thereby contribute to the improvement of lipid accumulation and cell injury [36]. In addition, PPARG activation has been associated with COX-2 suppression in HepG2 cells [37], raising the possibility that PPARG restoration and PTGS2 inhibition are functionally connected. Nevertheless, the present findings do not establish whether PPARG acts upstream of PTGS2 or whether these pathways operate in parallel. Thus, PPARG may primarily contribute to the restoration of metabolic and antioxidant homeostasis, whereas PTGS2 may participate in the regulation of inflammatory lipid signaling.

In conclusion, this study is the first to demonstrate that xanthoxylin exerts potent protective effects against ALI, including alleviation of ethanol-induced cellular damage, reduction in TC and TG accumulation, attenuation of mitochondrial ROS production, and enhancement of SOD activity. These effects are likely mediated through the EGFR/AKT pathway. While several limitations should be acknowledged, our findings provide a strong rationale for further investigations, including surface plasmon resonance binding assays, primary hepatocyte models, pharmacokinetics, toxicity evaluation, and in vivo validation. Collectively, xanthoxylin emerges as a promising lead compound for ALI therapy.

## 4. Materials and Methods

### 4.1. Materials

HepG2 cells were purchased from the Kunming Cell Bank of the Chinese Academy of Sciences (Kunming, China). Xanthoxylin was purchased from Yuanye Biotechnology Co., Ltd (Shanghai, China). Fetal bovine serum (FBS) was purchased from Grand Island Biological Co., Ltd (Grand Island, NE, USA). Dulbecco’s Minimal Essential medium was purchased from Dalian Meilun Biotechnology Co., Ltd (Dalian, China). Penicillin/streptomycin (P/S), Trypsin-EDTA solution, RIPA lysis buffer, 30% Acrylamide Gel Solution, and methyl thiazolyl tetrazolium (MTT) were purchased from Solarbio (Beijing, China). MitoSOX™ Red Mitochondrial Superoxide Indicator was purchased from Thermo Fisher Scientific (Shanghai, China). The antibodies against p-EGFR, EGFR, p-Akt, Akt, PPARG, PTGS2, and β-actin were purchased from ABclonal (Wuhan, China). HSP90 antibody was purchased from Proteintech (Wuhan, China). Total cholesterol assay kit, triglycerides assay kit, and SOD assay kit were obtained from Nanjing Jiancheng Bioengineering Institute (Nanjing, China).

### 4.2. Collection of Xanthoxylin Targets and Gene Targets Related to Alcoholic Liver Injury

The PubChem database (https://pubchem.ncbi.nlm.nih.gov/, accessed on 26 May 2026) was utilized to obtain the SMILES structure of xanthoxylin. The targets of xanthoxylin for which the probability was >0 were obtained from the Swiss Target Prediction (https://swisstargetprediction.ch/, accessed on 26 May 2026), the SMILES structure of xanthoxylin was imported and the species was set as “Homo Sapiens”. Potential targets of alcoholic liver injury were obtained from the GeneCards disease database (https://www.genecards.org/, accessed on 11 May 2026). In order to explore the potential targets through which xanthoxylin acts on alcoholic liver injury, SangerBox (http://sangerbox.com/, accessed on 26 May 2026) was used to analyze the intersected targets of xanthoxylin and alcoholic liver injury.

### 4.3. Analysis of Protein–Protein Interaction (PPI) Network

The potential targets of xanthoxylin for ameliorating alcoholic liver injury were imported into the STRING analysis platform (https://cn.string-db.org/, accessed on 26 May 2026). Then, the organism was set as “Homo sapiens”, while the minimum medium confidence was set to 0.40. After obtaining the PPI network relationship data, the PPI network was rendered with Cytoscape 3.10.4 software.

### 4.4. GO Enrichment and KEGG Pathway Enrichment Analysis

To predict biological process (BP), cellular component (CC), molecular function (MF) and the signaling pathway associated with xanthoxylin’s mitigation of the symptoms of alcoholic liver injury, the GO enrichment and KEGG pathway enrichment analyses were conducted. The putative therapeutic targets of xanthoxylin for alcoholic liver injury were loaded into DAVID database, and “Homo sapiens” was chosen as the research taxonomy to conduct enrichment analysis. Enrichment analysis was performed in DAVID with default parameters (EASE threshold = 0.1, gene count threshold = 2). The *p*-value cutoff was set as 0.01. Terms were sorted by ascending *p*-value, and the top 10 entries were selected for GO and KEGG visualization of enrichment analysis via an online platform for data analysis and visualization (https://www.bioinformatics.com.cn/, accessed on 26 May 2026).

### 4.5. Molecular Docking

The SDF format of xanthoxylin was obtained from the PubChem database (https://pubchem.ncbi.nlm.nih.gov/, accessed on 29 May 2026). The three-dimensional protein structure models of BCL2 (PDB ID: 5UUK), EGFR (PDB ID: 8A27), ESR1 (PDB ID: 1XPC), GSK3B (PDB ID: 1Q41), HSP90AA1 (PDB ID:3T0H), PPARG (PDB ID: 8B94), HIF1A (PDB ID: 4H6J), and PTGS2 (PDB ID: 5IKR) were obtained from the PDB database (https://www.rcsb.org/, accessed on 29 May 2026). Subsequently, AutoDock Tools 1.56 was used to remove water molecules, add hydrogens, assign Gasteiger charges, and define the grid box for these protein models. The centers of grid used for the docking search were determined based on the literature [38,39,40,41,42,43,44,45]. Molecular docking was conducted via AutoDock vina 1.2.7, and the best poses with lowest binding energy were selected to further analyze and visualize the ligand–receptors interactions by DiscoveryStudio 2024 and PyMol 2.6.0 software.

### 4.6. Molecular Dynamics Simulation

The best docking configurations between xanthoxylin and target proteins, obtained from molecular docking, were utilized for molecular dynamics (MD) simulations using the GROMACS 2020.6 package. The force field of AMBER99SB and the SPC water model were applied to parameterize the key target proteins, while the ligand was generated with the sobtop_1.0 (dev3.1) tool based on the GAFF2 force field. To create the simulation system of these complexes, a cubic box with a minimum distance of 1.0 nm from the box edges was generated to enclose these receptor proteins, including EGFR, PTGS2, and PPARG, and the net charge of the ligand–receptor complexes was neutralized by adding counterions (Na^+^ or Cl^−^). Subsequently, in order to optimize the atomic positions, energy minimization was performed using the steepest descent and conjugate gradients methods, with 5000 steps, respectively. Then, equilibrium simulations lasting 500 ps were conducted under both normal volume temperature (NVT) and normal pressure temperature (NPT) conditions. Temperature was maintained at 300 K using a Nosé–Hoover thermostat, and pressure was kept at 1 bar with a Parrinello–Rahman barostat. Finally, the 200 ns MD program was run with a 2 fs integration time step. The root mean square deviation (RMSD), radius of gyration (Rg), solvent accessible surface area (SASA), root mean square fluctuation (RMSF), and hydrogen bond number were analyzed, thereby evaluating the stability and structure changes in the system.

### 4.7. Cell Culture

HepG2 cells were seeded in a 100 mm culture dish and cultured in DMEM medium supplemented with a mixture of 10% fetal bovine serum (FBS) and 1% streptomycin (P/S). The cells were placed in a humidified incubator at 37 °C and 5% CO_2_, and the culture medium was changed every two days; when the cell confluence reached 85% to 90%, the subsequent experiment begun.

### 4.8. MTT Assay

HepG2 cells were seeded into a 96-well plate at a density of 2 × 10^4^ cells and cultured for 24 h with different concentrations of xanthoxylin (5, 10, 20, 40, 80, 160 μM). A total of 20 μL MTT solution was added into each well and incubated in the dark for 4 h. Then, 200 μL of dimethyl sulfoxide (DMSO) was added, the mixture was shaken at room temperature for 10 min, and the absorbance was measured at 492 nm.

After determining the concentration of xanthoxylin, HepG2 cells were treated with 400 mmol/L ethanol and different concentrations of xanthoxylin for 24 h. After further cultivation in the incubator for 24 h, the MTT assay was used to determine the effects of ethanol and xanthoxylin on HepG2 cells.

### 4.9. Total Cholesterol and Triglyceride Testing

HepG2 cells were seeded in 60 mm culture dishes at a density of 2 × 10^6^ cells per dish. Cells were treated with 400 mmol/L ethanol and different doses of xanthoxylin for 24 h. Subsequently, protein extraction was performed on the cells using cell lysate. According to the instructions, a total cholesterol assay kit and a triglycerides assay kit were used to test the prepared protein extract, and finally measure the absorbance value using FlexStation 3 microplate reader (Molecular Devices, Shanghai, China).

### 4.10. Detection of Mitochondrial Reactive Oxygen Species (ROS) and SOD Activity

HepG2 cells were seeded into 12-well plates containing sterile coverslips at a density of 1 × 10^5^ cells per well. Following a 24 h incubation, the culture medium was replaced with serum-free medium supplemented with ethanol and varying concentrations of xanthoxylin, and the cells were incubated for an additional 24 h. Subsequently, the supernatant was aspirated, and the cells were gently washed before being incubated with 5 µM MitoSOX Red dye (Thermo Fisher Scientific, Shanghai, China) in fresh medium at 37 °C for 30 min. After incubation, the cells were washed twice with phosphate-buffered saline (PBS) and counterstained with Hoechst 33342 solution (Beyotime, Shanghai, China) for 10 min to label the nuclei. The coverslips were then carefully removed from the wells, gently inverted, and mounted onto glass slides using a drop of glycerol-based mounting medium. Fluorescence images were captured using an inverted fluorescence microscope.

HepG2 cells were seeded into a 96-well plate at a density of 2 × 10^4^ cells per well. After a 24 h incubation, the cells were treated with 400 mmol/L ethanol together with 40 or 80 μM of xanthoxylin for an additional 24 h. Finally, the SOD activity of HepG2 cells was determined using the corresponding assay kit.

### 4.11. Western Blotting

HepG2 cells were seeded in 60 mm culture dishes at a density of 2 × 10^6^ cells per dish and treated with ethanol and varying concentrations of xanthoxylin for 24 h. Total proteins were extracted using RIPA lysis buffer supplemented with phenylmethylsulfonyl fluoride (PMSF) at a ratio of 100:1 (*v*/*v*). Equal amounts of protein were separated by sodium dodecyl sulfate–polyacrylamide gel electrophoresis (SDS-PAGE) and subsequently transferred onto polyvinylidene difluoride (PVDF) membranes. The membranes were blocked with 5% (*w*/*v*) skim milk in Tris-buffered saline containing 0.1% Tween-20 (TBST) for 1 h at room temperature. Thereafter, the membranes were incubated overnight at 4 °C with primary antibodies against p-RGFR, EGFR, p-Akt, Akt, HSP90, PTGS2, PPARG, and β-actin, followed by incubation with horseradish peroxidase (HRP)-conjugated secondary antibodies for 1 h at room temperature. Protein bands were visualized and imaged using the FluorChem E System (Santa Clara, CA, USA).

### 4.12. Statistical Analysis

Statistical analysis was conducted using Graphpad Prism 10.0 software. All experiments were conducted with at least three independent biological replicates. The data are presented as mean ± SEM. Comparisons among multiple groups were conducted using one-way analysis of variance (ANOVA), followed by Duncan’s multiple test for pairwise comparisons. Statistical significance was set at *p* < 0.05.

## 5. Conclusions

In summary, this study suggests that xanthoxylin may be a promising hepatoprotective agent against alcoholic liver injury. The molecular mechanism involves the EGFR/AKT pathway, along with PPARG activation and PTGS2 inhibition. The integrated in silico–in vitro strategy exemplifies a rational framework for natural product mechanism elucidation, potentially accelerating the discovery of novel ALD therapeutics.

## Data Availability

The original contributions presented in this study are included in the article. Further inquiries can be directed to the corresponding authors.

## Abbreviations

The following abbreviations are used in this manuscript:

ALD	Alcoholic liver disease
ALI	Alcoholic liver injury
GO	Gene ontology
KEGG	Kyoto Encyclopedia of Genes and Genomes
EGFR	Epidermal growth factor receptor
Akt	protein kinase B
PPARG	Peroxisome proliferator-activated receptor gamma
PTGS2	prostaglandin-endoperoxide synthase 2
MTT	Methyl thiazolyl tetrazolium
SDS-PAGE	Sodium dodecyl sulfate-polyacrylamide gel electrophoresis
PPI	Protein–protein interaction
DMSO	Dimethyl sulfoxide
MD	Molecular dynamics
RMSD	Root-mean-square deviation
Rg	Radius of gyration
SASA	Solvent-accessible surface area
RMSF	Root-mean-square fluctuation
ROS	Reactive oxygen species
